# Responsive Complementary Feeding Practices in Rural Muhanga District of Rwanda: A Mixed Methods Study

**DOI:** 10.1002/puh2.206

**Published:** 2024-07-02

**Authors:** Jeanine Ahishakiye, Lenneke Vaandrager, Eric Matsiko, Philemon Kwizera, Maria Koelen

**Affiliations:** ^1^ Human Nutrition and Dietetics Department College of Medicine and Health Sciences University of Rwanda Kigali Rwanda; ^2^ Health and Society Chair Group Wageningen University and Research Wageningen The Netherlands

**Keywords:** infant feeding, mother‐child interaction during feeding, responsive feeding, Rwanda

## Abstract

**Background:**

Responsive feeding among infants and young children (IYC) determines their food acceptance and adequate dietary intake, which enhances growth and developmental opportunities. However, little is known about responsive feeding practices among IYC in Rwanda. This study explored the status and barriers of responsive feeding practices among mothers from rural areas of Muhanga District in Rwanda.

**Method:**

This descriptive longitudinal and exploratory mixed methods study was conducted among 29 mothers from 2 rural health centers in Muhanga District. Mothers were interviewed, and their interactions with children during lunch meals at 6, 9, and 12 months were observed. The interviews were recorded, transcribed, and thematically analyzed using Atlas.ti software.

**Results:**

The study shows that the number of mothers who reported to verbally encourage their children to eat during feeding increased with the child's age. Less than half of mothers, at all three time points of data collection (at 6, 9, and 12 months), reported and were observed allowing their children to self‐feed, smiling and talking to them during feeding. The perceived major barriers to mother–child interaction during feeding were lack of time due to the burden of other responsibilities, and poverty. In addition, fear of messing up and food waste were barriers to child self‐feeding opportunities.

**Conclusion:**

Findings indicate that responsive feeding was less practiced among study mothers due to lack of time, poverty, and fear of food waste during child self‐feeding. Nutrition interventions in this community should consider raising awareness of responsive feeding practices through education and encouraging mothers to devote sufficient time to interact with their children during feeding episodes.

## Graphical abstract

This study revealed that less than half of mothers, at all three time points of data collection (at 6, 9, and 12 months), reported and were observed allowing their children to self‐feed, smiling, and talking to them during feeding. The study also found that the perceived major barriers to mother–child interaction during feeding were lack of time due to other responsibilities, poverty, and concerns about mess and food waste.

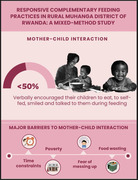

**Abbreviations**
IRB: Institutional Review Board
RDHS: Rwanda Demographic and Health Survey
WHO: World Health Organization

## 1 Background

Child undernutrition remains a public health concern in low‐ and middle‐income countries. It has long been documented that one of the major causes of child undernutrition relates to inadequate infant and young child feeding practices [1]. Optimal feeding practices not only depend on what and when to feed the child (the quality, the quantity, and the frequency) but also on how the child is fed and the quality of interaction between the mother and the child during feeding [2]. Undernutrition may be due as much to difficulties in the interactions between mothers and children as to a lack of high‐quality foods [3]. The interactions between mother and child that lead to a positive feeding experience, adequate dietary intake, and enhanced developmental opportunities are referred to as “responsive feeding” [4]. Preferably, children should be fed responsively [5].

Components of the responsive feeding practices as recommended by the Word Health organization (WHO) include (1) feeding infants directly and assisting older children when they feed themselves, being sensitive to their hunger and satiety cues; (2) feeding slowly and patiently and encouraging children to eat, but not forcing them to eat; (3) trying other encouragement strategies when children refuse food, and experimenting with different food combinations, tastes, textures, and methods of encouragement; (4) minimizing distractions during meals; and (5) remembering that feeding times are periods of learning, love, and talking to children during feeding with eye‐to‐eye contact [5].

Responsive feeding practices, as recommended by the WHO [5], are known to promote children's physical, mental, and social development [6]. Studies in low‐ and middle‐income countries showed that responsive feeding practices during the complementary feeding period increased child acceptance of food [3, 7, 8], adequate dietary intake, and good nutrition status [9, 10]. However, most of these studies were carried out in Asian countries, and very little research is available that documents responsive feeding practices in the African context.

Rwanda has made progress in decreasing the prevalence of acute malnutrition. However, the rate of chronic malnutrition (stunting) remains high (33%) particularly in rural areas [11]. Stunting reaches the highest point during the complementary feeding period. Adequate complementary feeding practices remain limited in Rwanda, as only 19% of children between 6 and 23 months of age met the minimum acceptable diet in 2019 [11]. In Rwanda, the efforts to improve complementary feeding have often focused on meal frequencies, dietary diversity, and composition [12], but very little on responsive feeding. Moreover, responsive feeding practices are less documented Rwandan. The present study aimed to explore the status and barriers of responsive feeding practices among mothers from rural areas of Muhanga District, Rwanda.

## 2 Methodology

### 2.1 Study Design

This study adopted a descriptive, longitudinal, and exploratory design using a mixed methods approach.

### 2.2 Study Setting

The study took place in rural areas of Muhanga District, located in the Southern province of the country, approximately 49 km from the Rwandan capital city of Kigali. The majority of the population in Muhanga District lives in rural areas, and agriculture is their main source of income. Muhanga District was selected based on its high rate of stunting among children aged 6–59 months (47%), food insecurity (26%), and poverty, with 56% of its population living below the poverty line [13, 14]. Particularly, the study was implemented in two governmental health centers (Buramba and Rutobwe) selected based on the stunting prevalence. Buramba Health Center has the highest rate of stunting in the district, and Rutobwe has the lowest rate of stunting.

### 2.3 Study Population and Recruitment

This study focused on mothers and their children aged 6–12 months. Mothers were enrolled during their last trimester of pregnancy in the larger longitudinal study that aimed to explore actual breastfeeding and complementary feeding practices among infants from birth to 1 year of age. The study was conducted during the period between December 2016 and October 2017. The study details and sampling procedures have been published elsewhere [15, 16].

Briefly, each pregnant woman in her last trimester of pregnancy who came for prenatal care consultation at Buramba and Rutobwe health centers during the selection period and who accepted to participate was enrolled on the study. Immediately after selection, for each participating pregnant mother, an in‐depth interview was conducted to explore prenatal infant feeding intentions, and the recruitment ceased after enrolling 39 women who came first as data saturation had been reached. Data saturation was set as a criterion to define the sample size. Data saturation was determined based on the fact that no new information was being obtained during the in‐depth interview with pregnant mothers. The inclusion criteria for the study were (1) being pregnant in the last trimester with no serious obstetrical condition, (2) planning to give birth in the local health center and reside in the area within the first 12 months of the child's life, and (3) willing to be observed during three child feeding occasions at 6, 9, and 12 months postpartum. Ten of the 39 recruited mothers were not considered in the current study because some were lost during the follow‐up (3 mothers were lost at 8 months), and others had no observation data (3 mothers at 8 months and 4 mothers at 12 months). The present study assessed only 29 mothers who completed the follow‐up from birth to 1 year of their child's life and whose data were available for both the interviews and mealtime observations.

### 2.4 Data Collection

Mother–child interactions at 6, 9, and 12 months of the child's life were assessed through in‐depth interviews and mealtime observations. First, in‐depth interviews were conducted, and then observations followed. To ensure the accuracy of the data, all in‐depth interviews and observations were collected by the first principal investigator with the help of one trained research assistant. The research assistant had a bachelor's degree in Human Nutrition, was experienced in qualitative data collection, and was trained on the tools and methods for 3 days. The training focused on research goals and objectives, how to collect data using an opportunistic observation form [17], the use of notebooks and forms to record observations in a systematic and organized way, how to conduct effective interviews, and ethical considerations. After training and before undertaking the data collection, interview and observation guides were reviewed and piloted among mothers who did not take part in the study. After piloting, the tools and guides were adjusted, where necessary.

#### 2.4.1 In‐Depth Interviews

Interview questions developed by the Pan American Health Organization and UNICEF [17] were adapted and used to collect data about responsive feeding practices. Table 1 summarizes the main content of the interview guide, which was administered to the mother. Questions asked the mothers how they fed their children during the main meal (lunch) the day before the interview. Mother encouragement during feeding was assessed by asking mothers if they did anything to encourage the child to eat and what they did, if mothers talked to the child during feeding and what they told their children, and how they managed food refusal. Mothers were also asked what they do if the child stops eating or when they feel their child is still hungry or has not eaten enough. Strategies to motivate the child to eat, and if not, the reasons for not motivating the child to eat or difficulties she would have in doing this were also a topic of the interviews. Mothers were also asked if the child was sometimes given the opportunity to self‐feed at any time during the feeding episode. At each follow‐up interview, the summarized key points from the previous interview were presented to the mother to confirm and establish continuity in her narratives and validate data from the previous interview [18]. For instance, at 9 and 12 months, the interviewer asked the mothers if they had made some changes in the ways they interacted with their babies during feeding from the last visit. If yes, mothers were invited to talk more about the changes they made and the reasons behind the changes. The in‐depth interviews were conducted in Kinyarwanda (mother tongue), and each interview lasted between 30 and 60 min.

**TABLE 1:** Interview guide at 6, 9, and 12 months among mothers from Muhanga District, Rwanda, 2017.

How did you interact with your baby during the main meal (lunch) yesterday? Probing questions 1.1. Yesterday, during mealtime, did you do anything to encourage (child's name) to eat? If yes, what did you do? If not, ask for reasons. Is it always like that, or are there situations where you do it differently? If yes, when and what do you do differently, and why? 1.2. Yesterday, during mealtime, while feeding (the child's name), did you talk to him/her? What did you say? Is it always like that, or are there situations where you do it differently? If yes, when and what do you do differently, and why? 1.3. Yesterday, during mealtime, did (the child's name) self‐feed (eat by himself/herself, using hands or utensils) at any moment during the meal? If yes, did the child (name) self‐feed the whole time, half of the time, or a little time? Is it always like that, or are there situations where you do it differently? What difficulties would you have in doing this? 1.4. Yesterday, during mealtime, did (the child's name) stop eating? If you think he/she did not eat enough, what did you do? Is it always like that, or are there situations where you do it differently? If yes, when and what do you do differently, and why? If the mother answers, “I motivated her/him to eat,” how did you motivate her/him to eat? Is it always like that, or are there situations in which you do it differently, and why? 1.5. What difficulties would you have in motivating the child to eat when you think he/she has not eaten enough? 1.6. If the mother does not say, she would motivate: Why would not you motivate? 1.7. What factors do you think make it difficult for you to interact with your child during feeding? How do they make your interactions difficult?

#### 2.4.2 Observation of Feeding Episode

At 6, 9, and 12 months postpartum, data were collected on mother–child interaction during feeding using a structured observation guide to generate a description of the daily life of mothers caring for their children in their naturalistic rural setting. Three lunchtime meal observations per child were made. The observations using opportunistic observation form (Table 2) were done when the principal investigator and the research assistant visited mothers to conduct in depth interviews.

**TABLE 2:** Observation guide with mothers from Muhanga District, Rwanda, 2017.

TOPIC
**1. Mother–child interactions (responsive feeding at 6, 9, and 12 months)**
1.1. Who is feeding the child during the mealtime? 1.2. What is the location of the mother in relation to the child during feeding? 1.3. Does the mother give the child the opportunity to self‐feed himself/herself during the feeding episode?
1.4. Does the child eat from his own plate or a shared plate with family members?
1.5. What kinds of foods are given to the child of about [child's age] months (foods, dishes, and drinks served to child)?
1.6. Does the mother serve additional portions to the child during the meal?
1.7. Does the child eat all of the food/drink he/she is served?
1.8. Does the mother talk to the child, verbally encouraging him/her to eat? What does the mother say?
1.9. Does the mother encourage the child when he/she is eating well? What does the mother do or say?
1.10. Does the mother ever motivate the child to eat more using gestures or games, singing, or demonstrating to her/him how to eat? What other strategies does the mother use?
1.11. Does the mother ever physically force the child to eat during the meal?
1.12. During the meal, does the child ever refuse the food? What does the mother do?
1.13. Is the child interested in food?

Three child practices were assessed: child's interest in food, self‐feeding attempts, and food refusal during the meal. A child was interested in food if he/she readily opened his/her mouth and moved his/her head toward the spoon or hands when food was offered, and not interested if he/she turned away from food every time he/she was offered food. Self‐feeding was defined as any bite a child attempted to feed himself/herself without assistance. Mothers’ practices that were assessed consisted of encouragement and strategies to overcome food refusal, if any. Encouragement was defined as verbally encouraging the child to eat when he/she is eating well and any nonaversive praise offered to the child by the mother, encouraging the child to eat more using gestures, games, or demonstrating how to eat. The mother's encouragement of self‐feeding was assessed by observing if the mother verbally encouraged, allowed, or supported the child to self‐feed himself/herself or to hold a spoon or touch food with her hand. Other aspects observed included who fed the child, what the child was given to eat, and where the feeding took place. In addition, any social interaction practice, verbal or gestural, which took place during feeding and concerned nonfood subjects was recorded. The observation was conducted from the start until the end of the mealtime.

The observers (the principal investigator and the research assistant) arrived at the participants’ homes in the morning and remained at the house until the lunch meal was prepared and the child had eaten to fill out the observation form. The date of the visit was not announced to the mothers, and the choice of food was free to ensure that mothers maintain their daily routine feeding practices.

### 2.5 Data Analysis

All in‐depth interviews were audio‐taped and transcribed verbatim in Kinyarwanda. Transcripts of interviews were made anonymous using participants’ codes. Analysis of in‐depth interviews was conducted using ATLAS.ti Software. Data analysis for in‐depth interviews followed the thematic analysis method, which involved reading the transcripts for familiarization with data, generating initial codes, sorting the different codes into potential themes, reviewing and refining themes, as well as reporting [19].

Initial codes were generated by the principal investigator using an inductive approach across the data set. Codes were reviewed and discussed by the first, second, and third authors. Then, the codes were sorted into themes, representing mother–child interaction during complementary feeding and barriers to mother–child interaction during complementary feeding. Themes were discussed in meetings with the coding team/authors until a consensus was reached on the key themes. For the analysis of mealtime observations, the number of times a single practice occurred was counted. An action or a practice that happened at least two times during the meal was considered, whereas practices that occurred less than two times were counted as absent [3]. Relevant verbatim quotes, translated from Kinyarwanda to English, were incorporated in the presentation of the results to help with data understanding.

The process of translating the observation form into Kinyarwanda followed the following steps: The first author performed the initial translation, focusing on rendering the content accurately and clearly while maintaining the intended meaning, and then another public professional, a bilingual, performed a translation from Kinyarwanda to English to see if there might be any discrepancies or misinterpretations that may have occurred during the forward translation. The English version was reviewed and discussed by the first and second authors. The back‐translated questions maintained their original meaning. Relevant verbatim quotes, translated from the original language, which is Kinyarwanda, to English, were incorporated in the presentation of the results. Quotations are tagged by participant identification (W‐1 to W‐29) and by the time the interview was conducted (interviews when the child was 6, 9, and 12 months were indicated by months 6, 9, and 12, respectively).

For the analysis of mealtime observations, the number of times a single practice occurred was counted. An action or a practice that happened at least two times during the meal was considered present, whereas practices that occurred less than two times were counted as absent [3]. No statistical test was performed because the sample size was too small.

### 2.6 Ethical Consideration

The study protocol was approved by the Institutional Review Board of the College of Medicine and Health Sciences (Approval notice: No. 058/CMHS IRB/2016) in Rwanda. Informed consent was obtained from each mother for participation in in‐depth interviews and lunchtime meal observations. Mothers were assured that their participation was voluntary and confidential and that they were free to withdraw from the study at any time.

## 3 Results

### 3.1 Characteristics of the Study Participants

As can be seen from Table 3, the mean age of our respondents was 33 years, with a standard deviation of 4.03. Twenty‐three were married. Twenty‐seven could read and write. Less than half of the participants (10) completed primary school education. The main occupation for all participants was agriculture. Most of the participants had one or two children.

**TABLE 3:** Characteristics of the study participants from Muhanga District, Rwanda, 2017.

Characteristics	Frequency
**Age of the mother (years) *n* = 29**	
<21	2
21–30	9
>30	18
**Marital status**	
With partner	23
No partner	6
**Ability to read and write**	
Yes	27
No	2
**Education level of the mother**	
Cannot read and write	2
Primary incomplete	14
Primary complete	10
Secondary incomplete	3
**Main occupation**	
Farming	29

### 3.2 Responsive Feeding Practices Status and Its Barriers

**FIGURE 1:**
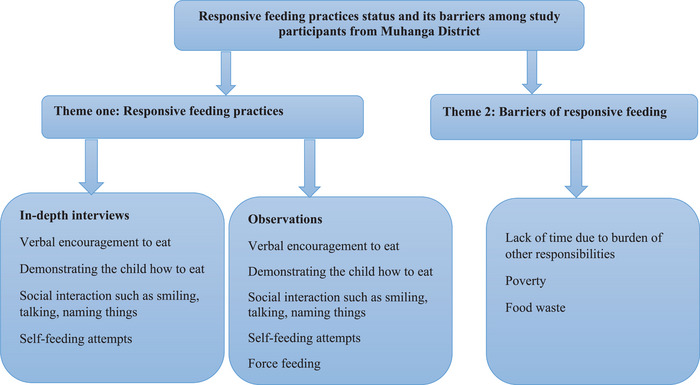
Responsive feeding practices and their barriers among study participants from Muhanga District, Rwanda, 2017.

The results of the interviews and observations were divided into two major themes: (1) mother–child interactions during complementary feeding episodes—responsive feeding practices, and (2) barriers to mother–child interactions during complementary feeding. These themes are outlined in a schematic diagram (Figure 1) of responsive feeding practices and their barriers.

### 3.3 Mother–Child Interaction During Complementary Feeding: Responsive Feeding Practices

During the observation at 6 months, the meal served to the child consisted mainly of cereal‐based gruel, a thin porridge commonly made from a mixture of two or more flours, such as maize, sorghum, and soybeans. At 9 and 12 months, the varieties of foods offered to children ranged from the aforementioned cereal‐based porridge to food from grain, root, and tuber groups (such as potatoes and plantains), legumes (mainly dried beans), and green leafy vegetables (amaranth).

Table 4 provides a brief description of the reported and observed practices during mealtime at 6, 9, and 12 months. The majority of mothers at all three time points (6, 9, and 12 months) reported verbally encouraging their children to eat, most commonly by making positive comments about food. However, at 6 months, some mothers pointed out that they did not see the importance of verbally interacting with such a young child during feeding. Those women highlighted that they did not understand why they would attempt to converse with a child who was still too young to talk.

**TABLE 4:** Reported and observed practices during mealtime at 6, 9, and 12 months among mothers from Muhanga District, Rwanda, 2017.

	6 months (*n* = 29)		9 months (*n* = 29)		12 months (*n* = 29)	
Actions	Reported	Observed	Reported	Observed	Reported	Observed
**Child's actions**						
*Interest in food*						
Interested	29	27	29	25	29	23
Not interested	0	2	0	4	0	6
*Self‐feeding*						
Self‐feeding attempt	0	0	4	0	10	13
**Mothers’ actions**						
*Strategies to encourage the child to eat*						
Verbal encouragement	17	1	22	13	23	14
Modeling eating/swallowing	7	8	2	5	0	0
*Encouragement of self‐feeding*						
Allow/support the child to self‐feed	0	0	7	0	12	13
*Social actions*						
Smiling/laughing	9	8	11	10	12	13
Talking but not about food (calling the child, naming things, asking a question)	0	0	9	6	11	8
*Violent behaviors*						
Force feeding by threating verbalization or physical force	0	2	0	3	0	3

At 9 and 12 months, many of the mothers verbally interacted with their infants. They described how they enjoyed dialogue (being talked to) and how their children ate a great amount of food if they were talked to during feeding. For instance, two mothers said:

I verbally encouraged him to eat by telling him that the food is very delicious which made him so happy that he ate the food with more interest. (W‐01, month 9)

Whenever I feed my kid I talk to him otherwise he doesn't eat. He looks aside in the opposite direction of the food even if he would still want more food. (W‐23, month 12)

Furthermore, at 9 and 12 months, some mothers explained that verbally encouraging the child during feeding promotes language development, on top of stimulating the child to eat more. For example, one mother said:

What I have observed is that when I talk to the child while eating, this motivates the child to eat more and boosts his capacity to speak. (W‐27, month 9)

Other mothers reported motivating the child to eat more by demonstrating to the child how to eat, whereas others reported engaging in social interactions unrelated to food during feeding episodes. Those nonfood‐related social interactions consisted mainly of drawing out a child's smile through copying and responding to the child's sounds and making funny sounds to get their child to smile, whereby the child responded with repetitive sounds (at 6 months). Mothers explained that drawing out a child's smile was used to make the child feel loved by the mother and that it promoted a bond between the mother and the child. Other mothers, at 9 and 12 months, reported talking to their children by calling the child's name or naming things. Some mothers explained that talking softly to the child was used as a way to provide social interaction, whereas others expressed talking to the child to assist the child with language development:

During feeding, I smile at him and see that the child is happy too. (W‐13, 6 months)

When I hold my child, I sometimes make funny sounds to get the child to smile, and see that the child feels loved. (W‐10, 6 months)

There is a time when he says anything and I respond by copying his sounds. By doing that, I realize that I stimulate the child's language development. (W‐8,12 months)

When he says anything, and I respond by copying the sounds, I see that the child is happy with that. (W‐27, 9 months)

The observation of feeding episodes showed that all infants were fed by their mothers and were given food from their plates. Most children were held by mothers, seated on mothers’ laps. Most children accepted food when it was offered. At 6 months, most mothers’ feeding practices consisted mainly of putting food into the child's mouth without any encouraging strategy or social interaction, whereas few mothers were observed demonstrating to the child how to eat. At 9 and 12 months, close to half of mothers were observed verbally encouraging their children to eat. In the case of food refusals during feeding, most participants reported encouraging their children to eat more. Observations, however, revealed that the majority of mothers did not try any solution in the case of food refusal but instead stopped feeding at all three time points. A few mothers were observed using threatening verbalization (giving direct orders) for more mouthful acceptance. For example, during the observation at 9 months, one mother said, “I will beat you if you don't eat this food. Eat.” Another mother was observed physically forcing the child to eat more by holding the child's hands down to force food into his mouth and commanding the child to eat. Table 5 summarizes the observed practices during mealtime at 6, 9, and 12 months.

**TABLE 5:** Observed practices during mealtime at 6, 9, and 12 months among mothers from Muhanga District, Rwanda, 2017.

Actions	6 months	9 months	12 months
**Child's actions**			
	Interest in food	Interest in food	Interest in food
		Self‐feeding	Self‐feeding
**Mothers’ actions**			
Verbal encouragement	Verbal encouragement	Verbal encouragement	Verbal encouragement
Role modeling of eating	Role modeling of eating	Role modeling of eating	
Allow/support the child to self‐feed		Allow/Support the child to self‐feed	Allow/Support the child to self‐feed
Smiling/laughing	Smiling/laughing	Smiling/Laughing	Smiling/Laughing
Talking to the child		Talking to the child	Talking to the child
Force feeding	Force feeding	Force feeding	Force feeding

At 6 months, most mothers reported feeding their infants and not allowing them to feed themselves. During the observation, at 6 and 9 months, all mothers fed their infants the entire time of the feeding episode, as they reported believing the children were still too young to feed themselves. At 12 months, almost half of the mothers provided their children with the opportunity to feed themselves small finger foods at some time during feeding. Those mothers who let their children self‐feed reported recognizing a child‐centered focus related to his/her development, learning, and autonomy, as two mothers stated:

I used to assist him in eating for the entire time of the feeding episode but later I decided to let him feed himself under my supervision as part of his learning process. For instance, I wash his hands and put the food on a small plate. Then the child starts eating himself. (W‐17 month 12)

I also assume that time will happen when I will not be nearby. Yet the child will have to eat. Consequently, I sometimes let him self‐feed under my supervision as the child needs to know how to feed himself as he grows older. (W‐12, month 12)

### 3.4 Barriers to Mother–Child Interactions During Complementary Feeding

At 6, 9, and 12 months, some mothers mentioned that the lack of time because of other responsibilities was a barrier to interact with their children during feeding. For instance, two mothers said:

I admit that this is a rare occasion where I talk to my child during feeding even though I know how important chatting is for the child but due to farm and household duties I do not do it either. (W‐25, month 6)

The child eats under pressure because I press him to finish the food quickly to let me go for my businesses, usually farming. Honestly, we do not have time to chat with the child during feeding. During working hours, I always put pressure the child to finish the food so that I get back to the farm, and searching for fodder but even at noon sometimes it becomes not possible because you are under pressure to be back to the farm. (W‐29, month 12)

Other mothers emphasized that poverty played a role in their inability to interact properly with children during feeding. Mothers said that their efforts were more focused on finding financial means to survive due to poverty.

It is just to find the means to survive due to poverty we only mind our own businesses. And if a mother spends her time conversing with her child, she can't achieve anything. She can't get anything to eat, as the family can't eat it. It is impossible. (W‐18, month 12)

Few mothers expressed (3 out of 29 at 12 months) their concern related to mess and food waste that may be associated with letting the child self‐feed, as two mothers explained:

She is too young to feed herself. If I let her instead she drops food on herself and her clothes and can be burnt. (W‐14, month 12)

The reason for not allowing the child to self‐feed is that the child does not value food, he throws them away rather than eating. (W‐7, month 12)

## 4 Discussion

The findings from the interviews and meal observations at 6, 9, and 12 months of child age indicated that the complementary foods offered to children were mainly starchy and leafy vegetables, whereas the consumption of animal‐source foods was limited. Similar findings were reported in other studies in Sub‐Saharan African countries [20, 21]. The consumption of animal‐source foods, including eggs and meats, is less common in most rural settings, yet these foods are beneficial to child growth and development due to their high content in proteins and micronutrients, which have been linked to improved nutritional status, including reduced stunting [22].

Our findings revealed that most mothers reported to verbally encourage the children to eat during feeding. However, this finding was not supported by the observation data, as most of the mothers at all three points (at 6, 9, and 12 months) were observed offering food to their children without any verbal encouragement. This discrepancy can be attributed to the fact that mothers are aware of the positive effects of verbal encouragement, yet they cannot manage to bring it into practice. This latter finding is consistent with the findings of another meal observation study in Kenya, which indicated that mothers were usually passive during meals [23]. However, positive verbalization during feeding is known to be important for greater food acceptance, a higher number of mouthfuls eaten, and good nutritional status in infants and young children [7, 24, 25]. Furthermore, positive mother verbalization during feeding provides a great opportunity to stimulate the child's social and mental development, promote psychosocial stimulation, and promote language and cognitive development in infants [24]. Therefore, the findings emphasize the need for practice‐change interventions encouraging mothers to combine feeding and stimulation activities for both better child nutrition and developmental outcomes. Such interventions may consider educating the mothers that, besides the satisfaction of child hunger, this moment is also crucial for helping children learn and show love by talking and maintaining visual contact with the child.

Mother–child interaction during feeding has been linked to the development of children's eating patterns and socialization [26]. Social interactions between mother and child, such as speaking to the child, singing, and encouraging him/her, also stimulate child's brain and promote cognitive development [27]. Therefore, nutrition interventions should support mothers in developing skills in specific forms of interaction during mealtime that promote full cognitive, physical, and social–emotional development in children.

The findings of this study also indicate that mothers’ feeding practices are age‐dependent. Over time, for instance, there was an increase (continuity) in mothers’ actions that included verbal encouragement, encouragement of self‐feeding, and social interactions such as talking to children from 6 to 12 months. This reflects that maternal feeding practices during infancy develop over time with respect to a child's developmental stages due to various factors, such as the child's nutritional needs and physical and cognitive development.

The findings also indicate that some mothers used negative strategies such as threatening verbalization during feeding, suggesting that mothers lacked problem‐solving strategies to keep on when feeding became challenging. Similar results have been described in other contexts, such as in Kenya [23] and Ghana [28]. The use of such negative strategies is an indication of nonresponsive feeding, which results in frequent child food refusals [29]. Negative feeding strategies, such as pressure, restriction, or verbal threats, are also recognized contributors to problematic eating behaviors, including aversions and disinterest in food [30]. The use of negative strategies has the potential to negatively impact child growth, particularly in communities where growth faltering is a major child nutrition problem.

Despite children's psychomotor ability to feed themselves from the age of 9 months [6], mealtime observations found that most mothers did not provide their children with the opportunity to self‐feed. Such nonresponsive feeding practices, characterized by the lack of adaptation to psychomotor abilities for self‐feeding, can negatively affect child feeding skills and healthy appetite in the long term [31]. A possible reason for not allowing their children to self‐feed may be that at that age, children need a long time to self‐feed, whereas mothers have no time due to competing demands from other responsibilities [32]. Another possible explanation may be the avoidance of food wastage, which is more likely to happen when the infant feeds herself/himself [33]. Nutrition interventions should consider training mothers on age‐appropriate, developmentally appropriate mother–child interaction and on developmental milestones related to feeding, such as praising the child for his ability to grasp small foods during the complementary feeding period. Mothers must be encouraged to allow their infant to self‐feed with age‐appropriate finger foods.

Lack of time, poverty, and fear of food waste were also barriers to responsive feeding practices. Due to other responsibilities, some mothers spend time on livelihood activities and household chores and, hence, have limited time to interact with children. This finding is similar to what has been reported by other studies conducted in other countries in Sub‐Saharan Africa [34, 35] and other developing countries such as Bangladesh [33]. The findings point out the need for interventions to encourage mothers to reallocate time to childcare or to integrate stimulation activities into their daily routines, as even a busy mother can be given the motivation and confidence to talk with a child during feeding [36]. An additional possible solution is to engage other family members, males included, in childcare practices to address the barrier of the burden of other responsibilities. This possible solution may also help to enhance the child's bonding with the father.

Lack of time becomes more critical in households in poverty, where mothers have to spend more time working for a living. Consistent with the literature, poverty and a lack of time have been cited as key factors that may disrupt mothers’ responsive feeding practices [33]. Moreover, poor households have limited resources, which limits self‐feeding practices among children because of concerns about food waste. This finding points out the need for interventions to encourage mothers to allocate sufficient time to childcare and interaction, especially during feeding, even when they are busy. Another possible solution is to engage other family members, fathers included, in childcare practices and spare mothers some of their responsibilities to address the burden of other responsibilities. Additionally, there is a need to support mothers in securing their income and access to food so that they can follow the responsive feeding recommendations. Such interventions may consider providing mothers with agricultural inputs and training, modern farming techniques, and income‐generating activities.

## 5 Strengths and Limitations

The strengths of this study lie in the use of a qualitative approach, which allowed to prospectively gain a deeper understanding of responsive feeding practices over time. The use of direct observation provided an opportunity to capture the verbal and nonverbal context of behavioral dynamics during feeding that was important yet not captured through self‐report interviews. Nevertheless, the study suffered from a number of limitations. First, mothers might have answered and modified their feeding practices as well as the way they communicate with their children in ways that they felt were more desirable during the observations due to our presence. We minimized reactivity by having established a sense of rapport and trust with mothers in the first 5 months of child's life during the exploration of exclusive breastfeeding practices. We believe that the effect of reactivity would have been minimal as mothers were exposed to repeated interviews and observations before they were recorded and observed (at birth, first week, 4 months). Second, because the study focused solely on a group of 29 mothers in rural Muhanga District of Rwanda, the findings have limited generalizability. Nevertheless, the primary objective of the study was not to ensure the generalizability of the findings. More research is needed to explore the association between behavioral factors in complementary feeding and children's nutrient intake.

## 6 Conclusion

This study finds that practices that include self‐feeding attempts, verbal encouragement, encouragement of self‐feeding, smiling, and talking to the child were less prevalent among participant mothers. Lack of time, poverty, and fear of food waste during self‐feeding were barriers to responsive feeding practices. Raising awareness of responsive feeding practices through education and encouraging mothers to devote sufficient time to interact with their children during feeding should be incorporated into any nutrition intervention in this study population.

## Author Contributions

J.A. designed the study protocol, conducted the in‐depth interviews, coded and analyzed the data, and wrote the manuscript. L.V. and M.K. contributed to the design of the study protocol, guided the analysis and writing of the manuscript, reviewed the manuscript, and approved it for submission. E.M. and P.K. contributed to the review of the manuscript. All authors read and approved the final manuscript.

## Conflicts of Interest

The authors declare no conflicts of interest.

## Data Availability

The data generated and analyzed during the current study are available from the corresponding author upon reasonable request.
